# Development of a bedside tool to predict the probability of drug-resistant pathogens among hospitalized adult patients with gram-negative infections

**DOI:** 10.1186/s12879-019-4363-y

**Published:** 2019-08-14

**Authors:** Thomas P. Lodise, Nicole Gidaya Bonine, Jiatao Michael Ye, Henry J. Folse, Patrick Gillard

**Affiliations:** 1 Albany College of Pharmacy and Health Sciences, Albany, NY 12208-3492 USA; 2 Allergan plc, Irvine, CA USA; 3 Allergan plc, Madison, NJ USA; 4 Evidera, San Francisco, CA USA

**Keywords:** Antimicrobial resistance, Gram-negative Bacteria, Prediction model

## Abstract

**Background:**

We developed a clinical bedside tool to simultaneously estimate the probabilities of third-generation cephalosporin-resistant Enterobacteriaceae (3GC-R), carbapenem-resistant Enterobacteriaceae (CRE), and multidrug-resistant *Pseudomonas aeruginosa* (MDRP) among hospitalized adult patients with Gram-negative infections.

**Methods:**

Data were obtained from a retrospective observational study of the Premier Hospital that included hospitalized adult patients with a complicated urinary tract infection (cUTI), complicated intra-abdominal infection (cIAI), hospital-acquired/ventilator-associated pneumonia (HAP/VAP), or bloodstream infection (BSI) due to Gram-negative bacteria between 2011 and 2015. Risk factors for 3GC-R, CRE, and MDRP were ascertained by multivariate logistic regression, and separate models were developed for patients with community-acquired versus hospital-acquired infections for each resistance phenotype (*N* = 6). Models were converted to a singular user-friendly interface to estimate the probabilities of a patient having an infection due to 3GC-R, CRE, or MDRP when ≥ 1 risk factor was present.

**Results:**

Overall, 124,068 patients contributed to the dataset. Percentages of patients admitted for cUTI, cIAI, HAP/VAP, and BSI were 61.6, 4.6, 16.5, and 26.4%, respectively (some patients contributed > 1 infection type). Resistant infection rates were 1.90% for CRE, 12.09% for 3GC-R, and 3.91% for MDRP. A greater percentage of the resistant infections were community-acquired relative to hospital-acquired (CRE, 1.30% vs 0.62% of 1.90%; 3GC-R, 9.27% vs 3.42% of 12.09%; MDRP, 2.39% vs 1.59% of 3.91%). The most important predictors of having an 3GC-R, CRE or MDRP infection were prior number of antibiotics; infection site; infection during the previous 3 months; and hospital prevalence of 3GC-R, CRE, or MDRP. To enable application of the six predictive multivariate logistic regression models to real-world clinical practice, we developed a user-friendly interface that estimates the risk of 3GC-R, CRE, and MDRP simultaneously in a given patient with a Gram-negative infection based on their risk (Additional file 1).

**Conclusions:**

We developed a clinical prediction tool to estimate the probabilities of 3GC-R, CRE, and MDRP among hospitalized adult patients with confirmed community- and hospital-acquired Gram-negative infections. Our predictive model has been implemented as a user-friendly bedside tool for use by clinicians/healthcare professionals to predict the probability of resistant infections in individual patients, to guide early appropriate therapy.

**Electronic supplementary material:**

The online version of this article (10.1186/s12879-019-4363-y) contains supplementary material, which is available to authorized users.

## Background

The prevalence of highly resistant Gram-negative infections has increased dramatically over the past decade among hospitalized patients throughout the United States [1] and the world [2]. Three of the more concerning antibiotic-resistant Gram-negative pathogens that are classified as serious or urgent threats to public health include third-generation cephalosporin-resistant Enterobacteriaceae (3GC-R), carbapenem-resistant Enterobacteriaceae (CRE), and multidrug-resistant *Pseudomonas aeruginosa* (MDRP) [1, 2].

Patients with infections due to 3GC-R, CRE, or MDRP have limited treatment options and are highly vulnerable to receiving inappropriate empiric or delayed appropriate therapy because clinicians frequently fail to recognize these infections prior to culture and susceptibility reporting. The deleterious consequences of delayed appropriate therapy are well described [3–10]. Not only does delayed appropriate therapy increase the risk of death [3, 5–10], but studies have also shown that delayed appropriate therapy prolongs hospitalization [3–10]. Prolonged hospitalization places patients at risk of developing subsequent antibiotic-resistant infections [3, 11], which can ultimately lead to further antibiotic usage [5] and exacerbate institutional antimicrobial resistance patterns [12, 13].

As a mechanism to promote appropriate antibiotic usage within a healthcare institution, the World Health Organization recommends that healthcare institutions create tools and implement policies informed by real-world data to increase the probability of patients receiving early appropriate therapy [14]. To enable the administration of early and appropriate therapy, it is important that patients at risk of being infected with resistant pathogens be identified promptly, especially as definitive culture results are typically not available within the first 24 to 72 h of infection onset [15, 16]. Although there is a general appreciation of patient-level risk factors (eg, comorbid conditions, immunosuppression) and exposures (eg, cumulative number of prior antibiotic exposures, prior hospitalizations) that increase the probability of having an infection due to 3GC-R, CRE, or MDRP, there are no widely available and readily adaptable tools for estimating the likelihood of having one of these resistant Gram-negative pathogens simultaneously in individual patients according to the presence or absence of critical hospital- and patient-level covariates.

To address this unmet need, the goal of this study was to develop a user-friendly clinical tool that simultaneously estimates the likelihood of having an infection due to 3GC-R, CRE, or MDRP when one or more risk factors are present among patients with infections due to Gram-negative bacteria. First, we sought to describe the risk factors for 3GC-R, CRE, and MDRP among patients with community- and hospital-acquired Gram-negative infections using data from a large cohort of hospitalized patients across multiple facilities. Prediction models were developed from these risk factor analyses to estimate the probability of having a 3GC-R, CRE, or MDRP infection when one or more risk factors were present in a given patient. The prediction models were then used to create a user-friendly clinical instrument for use at the bedside.

In summary, our prediction tool was developed to help clinicians identify hospitalized adult patients with Gram-negative bacteria who would benefit the most from tailored empiric treatment regimens in the critical period when a Gram-negative pathogen is identified on a Gram stain or with a rapid diagnostic test and antibiotic susceptibility results are not yet available. With this information, physicians can make more informed empirical antibiotic selections, and thereby increase the likelihood of timely appropriate antibiotic therapy. Studies have shown that the critical window between infection onset and delivery of appropriate antibiotics is 48–72 h [17–19]. Our tool aids in antibiotic selection during this critical time window as Gram stain and rapid diagnostic test results become available within the first 12–24 h of culture collection.

## Methods

### Data source

Input data for the development of the models were from a retrospective observational study of the Premier Hospital database, which contains coding and billing information for approximately 50 million admissions from more than 500 acute-care hospitals and is the largest hospital-based database in the United States. The study was limited to the approximately 160 institutions that contributed microbiology data for the entire study period of January 1, 2011, to October 1, 2015 [20]. The database was fully de-identified and compliant with the Health Insurance Portability and Accountability Act of 1996 (HIPAA); as such, no special permission was required to review patient records and extract the data. Given the de-identified and retrospective nature of the data, as well as the observational study design, written patient consent was neither required nor sought.

Patients included in the study population were adults (≥18 years) who had ≥1 admission to a hospital with evidence of a complicated urinary tract infection (cUTI), complicated intra-abdominal infection (cIAI), hospital-acquired or ventilator-associated pneumonia (HAP/VAP), or bloodstream infection (BSI) (Additional file 2). The index culture was defined as the earliest culture drawn that produced a positive finding for any Gram-negative bacteria. In addition, patients had to have a positive culture for Gram-negative bacteria drawn from a site consistent with the infection type (Additional file 2). Finally, patients were also required to have evidence of treatment with an intravenous antibiotic on the day of Gram-negative index culture collection or 2 days thereafter.

Infection groups were defined based on prespecified selection algorithms using primary or secondary International Classification of Diseases, Ninth Revision (ICD-9) diagnosis and procedure codes for each cohort (Additional file 2). Because codes were not mutually exclusive, an individual patient could contribute to more than one infection category during the study period.

3GC-R were defined as Enterobacteriaceae that were not susceptible to third-generation cephalosporins. CRE was defined as Enterobacteriaceae that were nonsusceptible to doripenem, meropenem, imipenem, or ertapenem. MDRP was defined as Pseudomonas that were not susceptible to at least three antipseudomonal agents, including penicillins, cephalosporins, monobactams, carbapenems, aminoglycosides, or fluoroquinolones. Nonsusceptibility was defined as either resistant or intermediate. Overall, susceptibility status was defined as infections that were susceptible to other antibiotics; patients who had an infection that could not be ascertained based on the susceptibility status were excluded from the study.

### Potential predictors

Potential predictors in the models included infection-, patient-, and hospital-level characteristics. Additional details related to the potential predictors in the models are found in Additional file 2. Infection-level characteristics included the site of infection, type of hospital unit (intensive care unit [ICU] vs non-ICU) at time of culture draw, or hospital- or community-acquired. The infection was considered to be hospital-acquired if the patient had an index culture date ≥3 days after hospital admission and was considered community-acquired for patients who had an index culture date < 3 days after hospital admission.

Patient-level characteristics included age, race, sex, marital status, and a composite comorbidity index (Charlson Comorbidity Index; eg, cancer, cerebrovascular disease, chronic pulmonary disease, congestive heart failure, diabetes with or without complications, dialysis, mild liver disease, myocardial infarction, paraplegia or hemiplegia, peripheral vascular disease) [21]. Patient-level characteristics also included admission type (emergency, urgent, elective, trauma center, or other), source (transfer, clinical referral, court/law enforcement, other, or unknown), prior all-cause hospitalization during the 6-month period before the admission, infections in the 3 months prior to the admission, and prior number of antibiotics. Prior antibiotic use was defined as use of antibiotic with activity against Gram-negative bacteria prior to index culture day in the qualified admission (Additional file 2). It was categorized as < 2, 2–3, and ≥ 4, indicating cumulative number of different antibiotics a patient received before index culture date in the qualified admission.

Hospital-level characteristics included the type of facility (ie, teaching vs nonteaching), setting (ie, urban or rural), geographic area (ie, Northeast, Midwest, South, or West), geographic division (ie, New England, Middle Atlantic, East North Central, West North Central, South Atlantic, East South Central, West South Central, Mountain, or Pacific), and number of beds (ie, 0–99, 100–199, 200–299, 300–399, 400–499, and ≥ 500). Prevalence of resistant Gram-negative infections (CRE, 3GC-R, and MDRP) in the hospital facility was included and defined by the median prevalence value across all hospitals.

### Statistical analyses

The first step of analysis was to summarize the study population by standard descriptive statistics (means for continuous variables, proportions for categorical variables) to compare patients with resistant and nonresistant infections. Pearson χ^2^ or Fisher’s exact test was performed for comparisons between two categorical variables; Student *t* or Mann-Whitney U test was used for comparisons between dichotomous and a continuous variable. Tests for trends were used to assess for the presence of a relationship between cumulative number of prior antibiotic exposures with Gram-negative activity and presence of resistant pathogen.

Because of the large number of predictors identified, least absolute shrinkage and selection operator (LASSO) logistic regression, or L1-regularized logistic regression, was used to select the set of predictors that best predicted the outcome by evaluating each coefficient simultaneously. Separate models for each resistance phenotype were conducted for hospital-acquired and community-acquired infections because they represent different patient populations with potentially different sets of risk factors for resistant infections. For the LASSO logistic regression analyses, the study sample was randomly split into the training set (70% of the study sample) and the test set (30% of the study sample). The training set was used to construct the LASSO logistic regression model. Cross-validation based on area under the curve (AUC) of receiver operating curves on training data was used to determine the best LASSO logistic regression model. Variables found to be predictive in the univariate analysis at *P* < 0.20 were included as potential predictors at model entry in each LASSO logistic regression analysis. Variables with *P* < 0.1 were retained in the final models. Model performance was evaluated using the model lift among the top 10% of scored subjects in the test data. The model lift was defined as the probability of a positive case given a top 10% score divided by the probability of a positive case in the overall sample. A higher lift indicated a stronger association between the predicted score and the outcome. The predicted and observed likelihood of each resistant phenotype in each model was examined for any discordance.

### Development of a user-friendly clinical bedside tool

Six models were developed: three for patients with hospital-acquired 3GC-R, CRE, and MDRP infections and three for community-acquired 3GC-R, CRE, and MDRP infections. A clinical prediction tool implemented using Microsoft Excel™ (Redmond, WA) was developed to provide a convenient interface to the statistical models for use at the patient bedside. Infection, hospital, and patient characteristics for an individual patient are input into the tool, which then displays the probability of a resistant infection in tabular and graphical forms. The final tool contains the hospital- and community-specific models for each category of resistant pathogens and is available from Additional file 1 together with a user guide.

## Results

### Study population and baseline event data

A total of 124,068 patients contributed to the dataset during the study period. Baseline event counts in the training and test datasets are shown in Additional file 2: Table S1. Numbers of patients admitted for cUTI, cIAI, HAP/VAP, and BSI were 76,367, 5649, 20,432, and 32,706, respectively (some patients contributed more than one infection type). The proportions of resistant infection in the study population overall were 12.09% for 3GC-R, 1.90% for CRE, and 3.91% for MDRP. Within each microbial category, a greater percentage of resistant infections were community-acquired relative to hospital-acquired (3GC-R, 9.27% vs of 12.09%; CRE, 1.30% vs 0.62% of 1.90% MDRP, 2.39% vs 1.59% of 3.91%.

### Bivariate risk factor analysis

Detailed results of the bivariate risk factor analyses are shown in Additional file 2: Table S2. The values in the table indicate the degree of increased risk conferred by having the level of the predictor indicated for that row. For yes/no predictors, a value of “no” was taken as the reference value and indicated no additional risk. A value of 0 for a predictor indicates that the predictor was not included in that model, based on the results of the multivariate analysis. Covariates remaining in the final models were then summarized in Table 1. The number of prior antibiotics received, infection site, infection during the previous 3 months, and hospital prevalence of the resistant pathogen were among the most important predictors across most of the six models (boldface cells in Table 1).

**Table 1:** Independent predictors of ≥1 type of resistant infection included in the multivariate analysis^a^

	Predictor	CRE		3GC-R		MDRP	
		Hospital	Community	Hospital	Community	Hospital	Community
Other	**Infection site** ^**b**^	✓	✓	✓	✓	✓	✓
	ICU vs non-ICU	✓		✓		✓	
	**Hospital prevalence**	✓	✓	✓	✓	✓	✓
Patient	**Age**			✓	✓		
	Transfer	✓	✓	✓	✓	✓	✓
	Admission in prior 6 months	✓	✓	✓	✓	✓	✓
	**Prior number of antibiotics** ^**c**^	✓		✓		✓	
	**Infection in prior 3 months**		✓		✓	✓	✓
Comorbidities^d^	Cancer			✓			✓
	Cerebrovascular disease	✓		✓			
	Chronic pulmonary disease					✓	✓
	Heart failure			✓			
	Diabetes with complications	✓		✓	✓		
	Diabetes without complications		✓		✓		✓
	Dialysis	✓	✓	✓	✓	✓	
	Mild liver disease	✓		✓			
	Myocardial infarction	✓				✓	✓
	Para/hemiplegia		✓		✓	✓	✓
	Peripheral vascular disease			✓			

*3GC-R* third-generation cephalosporin-resistant Enterobacteriaceae, *CRE* carbapenem-resistant Enterobacteriaceae, *ICU* intensive care unit, *MDRP* multidrug-resistant *Pseudomonas aeruginosa*

^a^Boldface cells = most important predictors across most models (predictors incurring the highest level of risk compared with the reference value).

^b^Complicated urinary tract infection, complicated intra-abdominal infection, bloodstream infection, or hospital-acquired/ventilator-associated pneumonia.

^c^Prior number of antibiotics was not included for community-acquired infection because of the difficulty of recovering accurate data

^d^Primary and secondary diagnoses were based on International Classification of Diseases, Ninth Revision, Clinical Modification [ICD-9-CM] codes

### Predictive models for hospital-acquired gram-negative infections

Among hospital-acquired infections, the most important predictor for all three resistant phenotypes was the number of antibiotics received during the current hospital admission. Infection type was predictive of the presence of resistant infection: cUTI was not a predictor of resistance, cIAI and BSI were predictive of CRE and 3GC-R only, and HAP/VAP predicted the presence of all three resistant phenotypes. Among the infection types examined, only HAP/VAP was a predictor of MDRP. Lesser predictors of CRE and 3GC-R infection, in addition to hospital prevalence, admitting source, and hospital admission during the previous 6 months (common to both hospital- and community-acquired infection mentioned above), were patient stay in the ICU and diabetes with complications. Prior infection in the last 3 months was predictive of MDRP infection, as were chronic pulmonary disease and paraplegia/hemiplegia. Comorbidities such as cancer, cerebrovascular disease, congestive heart failure, and mild liver disease predicted the hospital acquisition of 3GC-R and to a lesser extent CRE, but not MDRP (Table 1, Additional file 2: Table S2).

### Predictive models for community-acquired gram-negative infections

Predictors of all three resistant phenotypes in community-acquired infections included cUTI, infection during the 3 months before hospital admission, presence of paraplegia/hemiplegia, and diabetes without complications. For CRE, the most important predictors were prevalence, prior infection in the last 3 months, and infection site. For 3GC-R, age, prevalence, and prior infection in the last 3 months were most important. For MDRP, infection site, paraplegia/hemiplegia, prior infection in the last 3 months, and prevalence were most important (Table 1, Additional file 2: Table S2).

### Evaluation of model performance

Of the six models, those for 3GC-R had the highest correct prediction rate for resistant infection for both hospital- and community-acquired infections (18.4 and 21.7%, respectively). The MDRP models had the highest AUC and the highest lift (indicating a stronger association between predicted score and outcome) for both hospital- and community-acquired infections. Because of the importance of prior antibiotic use, the hospital-acquired models had higher AUC and lift values than the corresponding community-acquired models (Table 2).

**Table 2:** Evaluation of model predictive performance

	CRE		3GC-R		MDRP	
Performance Metric^a^	Hospital	Community	Hospital	Community	Hospital	Community
AUC for training data	0.90	0.79	0.87	0.71	0.94	0.84
AUC for test data	0.92	0.79	0.87	0.70	0.94	0.83
Correct prediction among top 10% scored subjects in test data, n/N (%)	145/3496 (4.15)	165/3607 (4.57)	627/3411 (18.38)	786/3624 (21.69)	405/3586 (11.29)	388/3628 (10.69)
Lift of top 10% scored subjects in test data^b^	7.8	3.8	6.4	2.6	7.9	5.0

*3GC-R* third-generation cephalosporin-resistant Enterobacteriaceae, *AUC* area under the receiver operating characteristic curve, *CRE* carbapenem-resistant Enterobacteriaceae, *LASSO* least absolute shrinkage and selection operator, *MDRP* multidrug-resistant *Pseudomonas aeruginosa*

^a^The method of predictor selection was LASSO logistic regression that minimized the cross-validation misclassification error; LASSO C value was 0.1

^b^Lift defined as probability of a positive case given a top 10% score divided by the probability of a positive case in overall sample. This ratio evaluates how much a top score enriches for selecting positive cases compared with random sampling in the absence of a model. A higher lift indicates a stronger association between the predicted score and the outcome

### Creation of a user-friendly Excel-based clinical bedside tool

To enable application of the six predictive multivariate logistic regression models to real-world clinical practice, we developed a user-friendly interface that estimates the risk of resistant infection. This tool is intended for use by clinicians at the patient bedside. Because it is not intended to be prescriptive, the tool simply estimates the risk of resistance without making specific recommendations regarding the best treatment. These recommendations will help the clinician select the most appropriate (empiric) antibiotic treatment for an individual patient according to the full context of patient characteristics and circumstances.

**Fig. 1:**
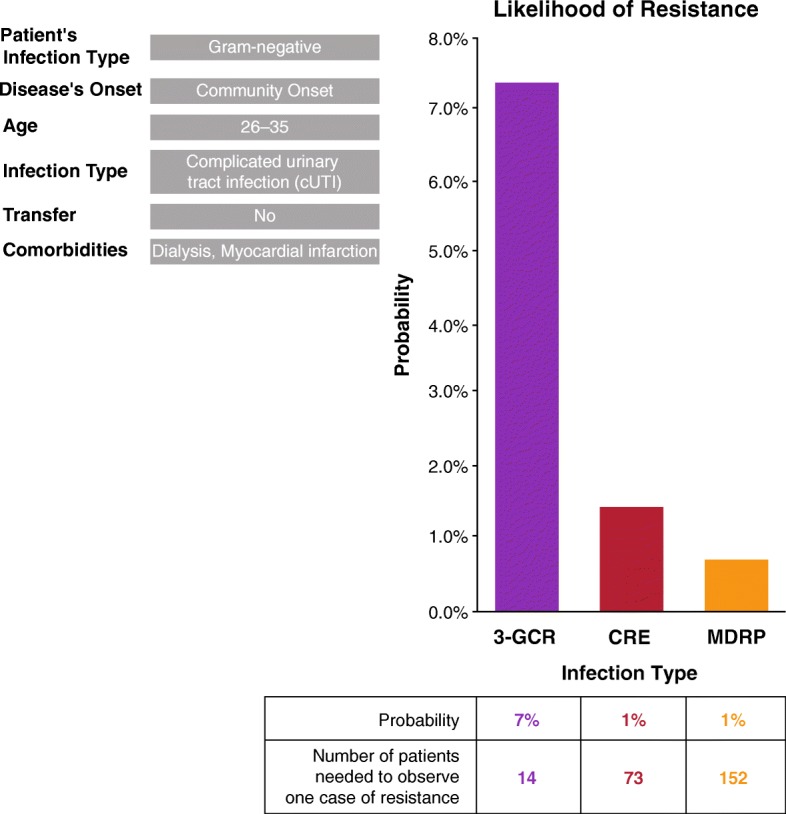
Clinical bedside tool to predict the probability of drug-resistant pathogens among an adult population with Gram-negative infections: sample result. 3GC-R = third-generation cephalosporin-resistant Enterobacteriaceae; CRE = carbapenem-resistant Enterobacteriaceae; MDRP = multidrug-resistant *Pseudomonas aeruginosa*

Consisting of four worksheets, the full tool and a detailed user guide are available at Additional file 1. User-modifiable drop-down lists are provided for entry of the model inputs of infection, hospital, and patient characteristics. A summary of the results is then provided, consisting of the risk factors and the likelihood of the presence of each type of resistant infection in the model, including the number of patients that need to be observed to detect one case of resistance (1/probability), which is easier to interpret in cases of small probabilities. A summary of the results for each run of the tool can be printed as a portable document format (PDF) report that includes the tool overview, model inputs, and results. In addition, a comprehensive calculation sheet can be accessed if required for more detailed data analysis (Fig. 1).

## Discussion

One of the fundamental pillars of antimicrobial stewardship is ensuring that patients with life-threatening infections receive early, appropriate antimicrobial therapy. Despite the longstanding recognition of the positive benefits of “getting it right the first time,” delayed appropriate therapy rates for patients with serious Gram-negative infections are still reported to be > 30% in several publications [3, 7]. To facilitate the administration of early appropriate therapy, it is important that patients at risk of being infected with resistant pathogens be identified promptly, especially as definitive culture results are typically not available within the first 24 to 72 h of onset of infection [15, 16].

Advances in rapid diagnostics have shortened the lag time between infection onset to pathogen identification from days to hours and have had positive effects on clinical outcomes when paired with robust antimicrobial stewardship programs [22]. Although rapid diagnostics represent a significant advance from traditional culture methods, current technologies are only able to identify a limited number of antibiotic-resistant Gram-negative pathogens. Therefore, clinical prediction tools, ideally in tandem with rapid diagnostic tests and Gram stain results, are needed to inform empiric antibiotic selection in the critical period when a Gram-negative pathogen is identified on a Gram stain or with a rapid diagnostic test and antibiotic susceptibility results are not yet available. To date, most published clinical prediction tools have focused on one pathogen or antibiotic-resistant phenotype [23, 24]. Although helpful in the antibiotic selection process, the risk factors for infections due to the various antibiotic-resistant Gram-negative pathogens are largely overlapping, and patients are at risk of several resistant Gram-negative pathogens simultaneously when a common risk factor, or combination of risk factors, are present. For example, prior receipt of carbapenems has been found to increase the risk of having an infection due to several highly resistant Gram-negative bacteria. Cognizant of this, we developed a clinical prediction tool that estimates the probabilities of having a Gram-negative infection due to 3GC-R, CRE, or MDRP. We selected these antibiotic-resistant Gram-negative pathogens because they are becoming increasingly common across most US healthcare institutions and are considered major threats to public health by the US Centers for Disease Control and Prevention [1].

When developing the clinical tool, we also believed it was important to develop two different models to differentiate the two distinct hospitalized patient populations with Gram-negative infections because risk factors or strength of their association can vary by population. The first focused on patients who presented to the hospital (ie, community-acquired); the second centered on patients who developed their infection during their hospitalization (ie, hospital-acquired). Interestingly, patients with community-acquired Gram-negative infections had a higher baseline risk of having resistant Gram-negative infections relative to those with hospital-acquired infections in this study, highlighting the need for the development of two separate clinical prediction models. Another distinguishing feature of this clinical prediction model was the inclusion of the prevalence of each resistant phenotype of interest in the hospital where the patient developed the infection. It is well established that a patient’s risk of having an antibiotic-resistant infection is driven in part by the bacteria present in the healthcare institution. Therefore, we believe it was important to consider it as a covariate in the clinical model-building phase. Finally, we believe that our model also rectifies issues related to autocorrelation, in which a patient could theoretically be counted twice if exposed to more than one type of infection. This exposure could potentially occur on different sites or on different days, leading to potentially different exposures. Because we considered the site of infection in model development, the risk of autocorrelation was minimized, confirming the quality of our model.

Although there were some distinguishing aspects to our clinical prediction tool, our model development approach was similar to previous studies [25–27]. Using the Premier Hospital database, which included 124,068 hospital admissions from approximately 160 institutions that contributed microbiology data during the period from January 1, 2011, to October 1, 2015, we first identified the infection-, patient-, and hospital-level risk factors that increase the probability of having an infection due to 3GC-R, CRE, or MDRP. Not surprisingly, risk factors and exposures associated with antimicrobial resistance in this study were largely consistent with previous reports [28–35]. The most important independent risk factors for both hospital and community acquisition of all three resistant bacteria were hospital prevalence of the resistant pathogen, admission source, and previous hospital admission within the prior 6 months [28–30, 32–35]. All three of these factors capture time at risk in healthcare facilities among patient populations predisposed to infection by antibiotic-resistant pathogens [28–35] and highlight the importance of understanding the prevalence of a given antibiotic-resistant phenotype in an institution. Another common risk factor for 3GC-R, CRE, and MDRP infection among individuals with hospital-acquired Gram-negative infections was the number of antibiotics received during the current admission [28–35]. Constant and cumulative exposure to antibiotics disturbs the natural bacterial flora, in particular in the gastrointestinal tract, and predisposes patients to colonization by resistant phenotypes. For all three pathogens, the presence of a resistant phenotype was predicted more strongly by previous use of four or more antibiotics than by previous use of two to three antibiotics in the current hospital admission. Our data suggest that a patient’s cumulative antibiotic exposure history is likely to be more important than any one specific antibiotic exposure when determining a patient’s likelihood of harboring a resistant pathogen.

The information from the multivariate logistic regression analyses was then used to develop models to estimate the likelihood of having these infections when one or more risk factors were present in hospitalized adult patients with Gram-negative infections. The major advantage of using logistic regression to develop clinical prediction rules is the functionality of the final models. In addition to identifying variables that are independently associated with stronger odds of having the outcome of interest, the final logistic regression models are mathematic equations that can be used to predict the probability of antimicrobial resistance based on the combination of significant risk factors present in a given individual with an infection [10, 36]. Adaptation of the models to provide a clinical tool was relatively straightforward because of their simplicity. With the information generated from this clinical prediction tool, we anticipate that clinicians will be able to make more informed empiric antibiotic selection decisions and thereby increase the likelihood of appropriate empiric antibiotic therapy. Although no specific recommendations are made regarding treatment options, the tool is designed as a simple interface to estimate the risk of resistance, which can be used by the clinician to determine the best course of treatment at bedside.

Several things should be considered when interpreting these findings. As the data used for the development of our models was from a database, our study is subject to the limitations associated with retrospective observations studies, and the ICD-9 codes may not be 100% accurate. As with all electronic health databases, there may be errors of omission and/or commission in coding. Because our operational definitions were based on information within the database, study measures may be less accurate than those based on medical record review or data gathered prospectively. Because the Premier Hospital database lacks information on healthcare utilization outside of Premier facilities, we did not include prior receipt of antibiotics in the community-acquired model. We did not consider prior colonization with a resistant pathogen as part of these analyses as only clinical culture data were available in the database. We also cannot exclude the possibility of patient-to-patient transmission of the resistant strains, which may weaken the association between acquisition of resistant pathogens and the identified risk factors. Another limitation was that the tool was not validated using an external dataset; a validation study should be performed*.* Finally, additional prediction methods such as neural networks, random forest, and SuperLearner, which allow for the incorporation of several algorithms simultaneously to deliver the strongest prediction model, may improve the prediction modeling observed in this study [37–39]. Despite these limitations, we think the model fit statistics demonstrate that we employed robust methodologies to derive the clinical prediction tool and adequately captured comorbid conditions, key baseline characteristics, and clinical covariates when deriving clinical prediction tools. More importantly, we believe our clinical prediction tool has merit, as it relied on the data elements that are typically available to the clinician at the time of empiric antibiotic selection among patients presenting with Gram-negative infections.

## Conclusions

Based on a large retrospective observational study, we developed six separate models for the prediction of hospital- and community-acquired infections due to 3GC-R, CRE, and MDRP among hospitalized adult patients with Gram-negative infections. The performance of our models is superior to or comparable with the performance of similar published models because of (1) the large number of patients and institutions contributing data, (2) the number and diversity of potential predictors considered, and (3) the inclusion of antibiotic resistance rates in the included hospitals. Our predictive model was implemented as a user-friendly bedside tool for use by physicians or healthcare professionals to predict the probability of resistant infection in an individual patient to expedite and direct initial antibiotic therapy and improve outcomes among hospitalized adult patients with Gram-negative infections in the critical period when a Gram-negative pathogen is identified on a Gram-stain or with a rapid diagnostic test and antibiotic susceptibility results are not yet available.

## Additional files

Additional file 1: contains the Clinical Bedside Tool. (XLSM 162 kb)

Additional file 2: contains Appendices A, B, and C, Tables S1, S2, and S3. (DOCX 51 kb)

## Data Availability

The data that support the findings of this study are available from Premier Hospital, but restrictions apply to the availability of these data, which were used under license for the current study and so are not publicly available. However, data are available from the authors upon reasonable request and with permission of Premier Hospital.

## Abbreviations

3GC-R: third-generation cephalosporin-resistant Enterobacteriaceae
AUC: area under the curve
BSI: bloodstream infection
cIAI: complicated intra-abdominal infection
CRE: carbapenem-resistant Enterobacteriaceae
cUTI: complicated urinary tract infection
HAP: hospital-acquired pneumonia
HIPAA: Health Insurance Portability and Accountability Act of 1996
ICD-9: International Classification of Diseases, Ninth Revision
ICU: intensive care unit
LASSO: least absolute shrinkage and selection operator
MDRP: multidrug-resistant *Pseudomonas aeruginosa*
ROC: receiver operating curves
VAP: ventilator-associated pneumonia
