# Supplementary material for: Development of a bedside tool to predict the probability of drug-resistant pathogens among hospitalized adult patients with gram-negative infections
Source: BMC Infect Dis. 2019 Aug 14;19:718. doi: 10.1186/s12879-019-4363-y (PMC6694572; doi:10.1186/s12879-019-4363-y)
Supplement: Supplementary file 2 — contains Appendices A, B, and C, Tables S1, S2, and S3. (DOCX 51 kb) [file 12879_2019_4363_MOESM2_ESM.docx]

Development of a Bedside Tool to Predict the Probability of Drug-Resistant Pathogens Among Hospitalized Adult Patients With Gram-Negative Infections

Running Title: Prediction of Drug-Resistant Gram-Negative Infections

Author(s): Thomas Lodise, Nicole Gidaya Bonine, Jiatao Michael Ye, Henry J. Folse, Patrick Gillard

**Supplemental Materials**

**Appendices**

**Appendix A**

**Complicated Urinary Tract Infection: ≥1 Diagnosis From Group A OR ≥1 Diagnosis From Group B AND ≥1 Event (Either Diagnosis or Procedure) From Group C**

|  | **Group A (Diagnosis Codes)** |
| --- | --- |
| **ICD-9-CM Diagnosis Code** | **Description** |
| 598.0X | Urethral stricture due to infection |
| 996.64 | Infection and inflammatory reaction due to indwelling urinary catheter |
| 590.1X | Acute pyelonephritis |
| 590.2X | Renal and perinephric abscess |
| 590.3X | Pyeloureteritis cystica |
| 590.8X | Other pyelonephritis or pyonephrosis, not specified as acute or chronic |
| 590.9X | Infection of kidney, unspecified |
| 590.XX | Infections of kidney |
| 590.0X | Chronic Pyelonephritis without Med Necr |
| 590.01 | Chronic Pyelonephritis with Med Necr |
| 590.1X | Acute Pyelonephritis NOS |
| 590.2X | Renal/Perirenal Abscess |
| 590.3X | Pyeloureteritis Cystica |
| 590.8X | Pyelonephritis NOS |
| 590.9X | Infection of Kidney NOS |

|  | **Group B (Diagnosis Codes)** |
| --- | --- |
| **ICD-9-CM Diagnosis Code** | **Description** |
| 598.8X | Other specified causes of urethral stricture |
| 598.XX | Urethral Stricture: infect NOS |
| 599.0X | Urinary tract infection, site not specified |
| 599.2X | Urethral Diverticulum |
| 599.3X | Urethral Caruncle |
| 599.4X | Urethral False Passage |
| 599.5X | Prolapse Urethral Mucosa |
| 599.69 | Urinary Obstruction NEC |
| 595.XX | Acute cystitis |
| 595.3X | Trigonitis |
| 595.4X | Cystitis in diseases classified elsewhere |
| 595.8X | Other specified types of cystitis |
| 595.9X | Cystitis, unspecified |
| 595.XX | Acute Cystitis |
| 595.1X | Chronic Interstitial Cystitis |
| 595.2X | Chronic Cystitis NEC |
|  |  |
| 595.81 | Cystitis Cystica |
| 595.82 | Irradiation Cystitis |
| 595.89 | Cystitis NEC |
| 595.9X | Cystitis NOS |
| 597.XX | Urethral abscess |
| 597.8X | Urethritis, unspecified |
| 597.80 | Urethritis NOS |
| 597.81 | Urethral Syndrome NOS |
| 597.89 | Urethritis NEC |
| 599.1X | Urethral Fistula |

| **Group C (Diagnosis and Procedure Codes)** | | |
| --- | --- | --- |
| **Code Type** | **Codes** | **Description** |
| ICD-9-CM procedure | 57.94 | Insertion of indwelling urinary catheter |
| ICD-9-CM procedure | 57.95 | Replacement of indwelling urinary catheter |
| ICD-9-CM procedure | 96.48 | Irrigation of indwelling urinary catheter |
| CPT-4 procedure | 51702 | Insertion of temporary indwelling bladder catheter, simple (eg, Foley) |
| CPT-4 procedure | 51703 | Insertion of temporary indwelling bladder catheter; complicated (eg, altered anatomy, fractured catheter/balloon) |
| ICD-9-CM diagnosis | V53.6X | Urinary catheter |
| ICD-9-CM diagnosis | V55.6X | Attention to other artificial opening of urinary tract |
| ICD-9-CM diagnosis | 592.XX | Nephrolithiasis or ureteric calculi |
| ICD-9-CM diagnosis | 753.2X | Obstructive defect of renal pelvis and ureter |
| ICD-9-CM diagnosis | 599.6x | Urinary obstruction |
| ICD-9-CM diagnosis | 600.XX | Prostate hyperplasia |
| ICD-9-CM diagnosis | 596.XX | Bladder neck obstruction |
| ICD-9-CM diagnosis | 591.XX | Hydronephrosis |
| ICD-9-CM diagnosis | 788.2X | Retention of urine |
| ICD-9-CM diagnosis | 596.4X | Atony of bladder |
| ICD-9-CM diagnosis | 596.53 | Paralysis of bladder |
| ICD-9-CM diagnosis | 596.54 | Neurogenic bladder |
| ICD-9-CM diagnosis | 596.55 | Detrusor sphincter dyssynergia |

**Complicated Intra-abdominal Infection: ≥1 Diagnosis From Group A AND ≥1 Procedure From Group B**

| **Group A (Diagnosis Codes)** | |
| --- | --- |
| **ICD-9-CM Diagnosis Code** | **Description** |
| 562.01 | Diverticulitis of small intestine (without mention of hemorrhage) |
| 562.03 | Diverticulitis of small intestine (with hemorrhage) |
| 562.11 | Diverticulitis of colon (without mention of hemorrhage) |
| 562.13 | Diverticulitis of colon (with hemorrhage) |
| 569.81 | Fistula of intestine, excluding rectum and anus |
| 569.82 | Ulceration of intestine |
| 569.83 | Perforation of intestine |
| 569.88 | Perforation of intestine |
| 531.1X | Gastric ulcer with perforation, acute |
| 531.2X | Gastric ulcer with hemorrhage and perforation, acute |
| 531.5X | Gastric ulcer with perforation, chronic |
| 531.6X | Gastric ulcer with hemorrhage and perforation, chronic |
| 532.1X | Duodenal ulcer with perforation, acute |
| 532.2X | Duodenal ulcer with hemorrhage and perforation, acute |
| 532.5X | Duodenal ulcer with perforation, chronic |
| 532.6X | Duodenal ulcer with hemorrhage and perforation, chronic |
| 533.1X | Peptic ulcer with perforation, acute |
| 533.2X | Peptic ulcer with hemorrhage and perforation, acute |
| 533.5X | Peptic ulcer with perforation, chronic |
| 533.6X | Peptic ulcer with hemorrhage and perforation, chronic |
| 534.1X | Gastrojejunal ulcer with perforation, acute |
| 534.2X | Gastrojejunal ulcer with hemorrhage and perforation, acute |
| 534.5X | Gastrojejunal ulcer with perforation, chronic |
| 534.6X | Gastrojejunal ulcer with hemorrhage and perforation, chronic |
| 540.0X | Acute appendicitis with generalized peritonitis |
| 540.1X | Acute appendicitis with peritoneal abscess |
| 541.XX | Appendicitis NOS |
| 542.XX | Other appendicitis |
| 567.XX | Peritonitis |
| 569.5X | Abscess of intestine |
| 572.XX | Abscess of liver |
| 575.4 plus 574.0, 574.3, 574.6, 574.8, or 575.0 | Acute cholecystitis* |
| 576.1X | Cholangitis |

*Patients who meet this criterion will need a minimum of two diagnosis codes.

| **Group B (Procedure Codes)** | | |
| --- | --- | --- |
| **Code Type** | **Codes** | **Description** |
| ICD-9-CM | 43.5X-43.9X, 44.40-44.42, 44.61, 45.6X-46.13, 46.20-46.23, 46.7X, 46.80-46.82, 46.91-46.94, 46.99, 47.XX, 50.0X, 50.12-50.69, 51.02-51.04, 51.13, 51.2X, 51.3X, 51.4X-51.5X, 51.61-51.63, 51.69, 51.7X, 51.81-51.83, 51.89, 51.91-51.95, 51.99, 52.01, 52.09, 52.12, 52.22, 52.3X-52.83, 52.92, 52.95, 52.96, 52.99, 54.1X, 54.21, 54.4X, 54.5X, and/or 54.92-54.95 | Laparotomy, laparoscopy, or percutaneous drainage |
| CPT-4 | 43117, 43118, 43121, 43122, 43631, 43632, 43633, 43634, 43775, 43845, 43620, 43621, 43622, 43840, 43500, 43501, 43502, 43840, 44602, 44603, 43840, 44110, 44111, 44120, 44121, 44125, 43496, 44120, 44121, 44125, 44126, 44127, 44128, 44132, 44133, 44202, 44203, 44660, 44661, 44132, 44133, 44155, 44156, 44212, 44143, 44144, 44160, 44204, 44205, 44206, 44141, 44144, 44155, 44156, 44160, 44204, 44205, 44206, 44212, 44146, 44147, 44207, 44208, 45395, 45397, 45550, 44139, 44140, 44144, 44147, 44213, 44660, 44661, 44210, 44211, 44212, 44137, 44150, 44151, 44155, 44156, 44157, 44158, 44620, 44625, 44626, 43845, 44160, 44144, 44145, 44146, 44799, 45113, 44211, 44141, 44188, 44320, 44322, 44604, 44605, 45820, 45825, 44300, 44322, 57307, 45562, 45563, 45800, 45805, 45126, 51597, 58240, 44120, 4412, 44125, 44150, 44155, 44156, 44187, 44310, 44144, 44187, 44310, 44151, 44316, 44799, 44210, 44211, 44212, 45136, 44602, 44603, 35870, 44640, 44650, 44602, 44602, 44615, 35780, 44640, 44650, 44604, 44605, 35870, 44650, 44370, 44379, 44383, 44397, 44602, 44603, 44604, 44605, 44615, 45327, 45345, 45387, 44799, 44050, 44055, 44799, 44137, 44139, 44213, 44700, 44701, 45136, 44970, 44950, 44955, 44960, 44900, 49406, 50845, 44650, 44799, 44979, 47010, 47001, 47100, 47560, 47561, 47700, 49220, 37200, 47561, 47300, 47120, 47122, 47125, 47130, 47133, 47140, 47141, 47142, 47380, 47381, 47382, 47399, 47120, 47122, 47125, 47130, 47140, 47141, 47142, 47133, 47140, 47141, 47142, 47350, 47360, 47361, 47362, 47350, 47360, 47361, 47362, 47490, 47480, 48000, 48001, 47480, 47560, 47561, 47600, 47605, 47600, 47605, 47610, 47612, 47620,47562, 47563, 47564, 47999, 47570, 47610, 47612, 47620, 47701, 47360, 47420, 47425, 47564, 47610, 47612, 47620, 47700, 47510, 47511, 43273, 47400, 47999, 48148, 47715, 47711, 47712, 47999, 47900, 47460, 47420, 47425, 47460, 47610, 47612, 47620, 47999, 47999, 43275, 47510, 47511, 49405, 48020, 48510, 48100, 48105, 48120, 48500, 43240, 48999, 48140, 48145, 48146, 48155, 48160, 48550, 48150, 48152, 48153, 48154, 48554, 43273, 48999, 48545, 48547, 48140, 48145, 48150, 48152, 48153, 48154, 48000,48001, 48556, 49000, 49220, 58960, 49002, 58960, 35840, 47015, 49020, 49040, 49060, 49412, 49320, 44820, 49203, 49204, 49205, 49215, 49255, 58662, 58943, 58950, 58951, 58952, 58953, 58954, 58956, 58957, 58958, 60545, 44180, 44005, 49402, 49324, 49325, 49418, 49419, 49421, 49999, 49425, 49426, 44050, 44055, 49412, 49418, 49419 | Laparotomy, laparoscopy, or percutaneous drainage |

**Hospital-acquired pneumonia, including ventilator-associated pneumonia (VAP)**

Algorithm 1

1. A primary OR secondary discharge diagnosis of VAP (997.31)
2. ≥1 positive culture for Gram-negative bacteria of interest drawn from a site consistent with pneumonia (the date of the earliest such culture will be deemed the “index date”)
3. Index date ≥3 days following admission; and
4. Receipt of antibiotics on the index date or within the 3-day period thereafter

Algorithm 2

1. Any discharge diagnosis (ie, principal or secondary) of pneumonia (excluding VAP) (481.xx, 482.xx, 483.xx, 484.xx, 486.xx, 482.0, 482.1, 482.2, 485.xx)
2. ≥1 positive culture for Gram-negative bacteria of interest drawn from a site consistent with pneumonia (the date of the earliest such culture will be deemed the “index date”)
3. Index date ≥3 days following admission; and
4. Receipt of antibiotics on the index date or within the 3-day period thereafter

Algorithm 3

1. Any discharge diagnosis (ie, principal or secondary) of pneumonia (excluding VAP)
2. ≥1 positive culture for Gram-negative bacteria of interest drawn from a site consistent with pneumonia (the date of the earliest such culture will be deemed the “index date”) <3 days following admission
3. Receipt of antibiotics on the index date or within the 3-day period thereafter; and
4. Evidence that the source of pneumonia was nosocomial (as opposed to community-acquired or healthcare-associated)

**Appendix B. Designated Culture Sites for Infection Types of Interest**

| **Culture Site** | **cUTI** | **cIAI** | **HAP** | **VAP** | **Bacteremia** |
| --- | --- | --- | --- | --- | --- |
| Urine culture | X |  |  |  |  |
| Gastric culture |  | X |  |  |  |
| Wound culture |  | X |  |  |  |
| Fluid culture |  | X |  |  |  |
| Body fluid culture |  | X |  |  |  |
| Wound culture deep |  | X |  |  |  |
| Aerobic culture |  | X |  |  |  |
| Tissue culture |  | X |  |  |  |
| Drainage culture |  | X |  |  |  |
| Wound culture aerobic/anaerobic |  | X |  |  |  |
| Wound culture aerobic |  | X |  |  |  |
| Wound culture superficial |  | X |  |  |  |
| Abscess culture |  | X |  |  |  |
| Blood culture |  |  |  |  | X |
| Quantitative blood culture |  |  |  |  | X |
| Respiratory culture |  |  | X | X |  |
| Quantitative respiratory culture |  |  | X | X |  |
| Respiratory culture-Cystic fibrosis |  |  | X | X |  |
| Sputum culture |  |  | X | X |  |
| Bronchial culture |  |  | X | X |  |
| Bronchial lavage/bronchoalveolar lavage |  |  | X | X |  |

**Appendix C. Gram-Negative Antibiotic Used Prior to Index Culture Day in the Qualified Admission**

Antibiotics used were paromomycin, sulfanilamide, amikacin, amoxicillin/clavulanic acid, amoxicillin, ampicillin/sulbactam, ampicillin, ampicillin/sulbactam, atovaquone, azithromycin, aztreonam, bacampicillin, carbenicillin, cefaclor, cefadroxil, cefamandole, cefazolin, cefdinir, cefditoren, cefepime, cefixime, cefonicid, cefoperazone, cefotaxime, cefotetan, cefoxitin, cefpodoxime, cefprozil, ceftaroline, ceftazidime, ceftibuten, ceftizoxime, ceftriaxone, cefuroxime, cephalexin, cefalotin, cephapirin, cephradine, chloramphenicol, cinoxacin, ciprofloxacin, clarithromycin, clindamycin, clofazimine, cloxacillin, colistimethate, daptomycin, demeclocycline, dicloxacillin, dirithromycin, doripenem, doxycycline, enoxacin, ertapenem, erythromycin, erythromycin/sulfisoxazole, fidaxomicin, gatifloxacin, gentamicin, imipenem, iodoquinol, kanamycin, levofloxacin, lincomycin, linezolid, lomefloxacin, loracarbef, meropenem, methicillin, metronidazole, mezlocillin, minocycline, moxifloxacin, nafcillin, nalidixic acid, neomycin, netilmicin, nitazoxanide, norfloxacin, novobiocin, ofloxacin, oxacillin, oxytetracycline, penicillin G, penicillin V, pentamidine, piperacillin, piperacillin/tazobactam, quinupristin/dalfopristin, sparfloxacin, spectinomycin, streptomycin, sulfadiazine, sulfamethizole, sulfamethoxazole, sulfasalazine, sulfisoxazole, telavancin, telithromycin, tetracycline, ticarcillin/clavulanic acid, ticarcillin, tigecycline, trimethoprim/sulfamethoxazole, tobramycin, trimethoprim, trimetrexate, troleandomycin, trovafloxacin, vancomycin, polymyxin B, and rifampin.

**Potential Predictors**

### **Patient-level characteristics**

Patient characteristics included age and sex; admission type (emergency, urgent, elective, trauma center, other); admitting source (transfer, clinical referral, court/law enforcement, other, or unknown); specialty of physician who ordered the culture; prior all-cause hospitalization during the 6-month period before admission; prior infections during 1, 3, and 6 months before admission; prior diagnosis and procedures in the 1, 3, and 6 months before admission; and hospital unit where index culture was collected (ICU vs general ward). The ICU date was defined as having ICU charge code before or at the same date of index culture. Prior antibiotic use was defined as use of antibiotic with activity against Gram-negative bacteria prior to index culture day in the qualified admission. It was categorized as 0, 1, 2, 3, 4, and ≥5, indicating cumulative number of different antibiotics a patient received before index culture date in the qualified admission. Infection type was also included. Each component of the Charlson Comorbidity Index was included only if it appeared in >5% of the sample population.

**Hospital-level characteristics**

Hospital characteristics included setting (rural vs urban), teaching facility (teaching vs nonteaching), geographic area (Northeast, Midwest, South, West), geographic division (New England, Middle Atlantic, East North Central, West North Central, South Atlantic, East South Central, West South Central, Mountain, Pacific), and number of beds. Prior antibiotic use at outside hospitals or in the outpatient setting was not collected because of the difficulty in recovering accurate data; thus, for the community-acquired cohort, prior antibiotic use was not assessed. Hospital-level characteristics also included the prevalence of resistant Gram-negative infections, CRE, 3GC-R, and MDRP, by year at included institutions.

The four models described above were stratified by hospital-acquired, community-acquired, and any onset infections because the treatment of these infections was different. If the index culture date was ≥3 days after admission, the infection was considered to be hospital-acquired. If the index culture date was <3 days after admission, the infection was considered to be presented at hospital admission and deemed community-acquired.

For Models 1 and 2, we included only hospitals that contributed microbiology data during the entire study period and had at least one CRE patient that met inclusion criteria.

- Model 1: Among patients with a confirmed Gram-negative infection, predict the probability of a CRE infection.
- Model 2: Among patients with a confirmed Enterobacteriaceae infection, predict the probability of a CRE infection.

For model 3, we included only hospitals that contributed microbiology data during the entire study period and had at least one 3GC-R patient that met inclusion criteria.

- Model 3: Among patients with a confirmed Gram-negative infection, predict the probability of an 3GC-R infection.

For Model 4, we included only hospitals that contributed microbiology data during the entire study period and had at least one MDRP patient that met inclusion criteria.

- Model 4: Among patients with a confirmed Gram-negative infection, predict the probability of an MDRP infection.

Microbiological data

Microbiological data included all designated positive clinical cultures for Enterobacteriaceae spp. Variables describing the susceptibility data included test method, result organism (specific bacteria tested for susceptibility), medication (list of antibiotics for which sensitivity testing was performed), result (result of test), and interpretation (categorical result of test).

# Statistical Analyses

Standard descriptive statistics were used to describe the study population and compare drug-resistant Gram-negative patients and all other patients. The same analysis was conducted for patients with Gram-negative resistant-specific pathogens. Continuous variables were expressed as means, and categorical variables were shown as counts and proportions.

### Univariate analyses

Univariate logistic regressions with subject random intercepts were used to evaluate which individual risk factors were predictive of (1) Gram-negative resistance among all patients with cultures drawn and (2) Gram-negative resistant-specific pathogens (CRE, 3GC-R, and CRP) among all patients with a Gram-negative infection.

### Multivariate analyses

All variables predictive of Gram-negative resistance and Gram-negative resistant-specific pathogens (CRE, 3GC-R, and MDRP) in the univariate analyses (*P*<0.2) were considered as potential predictors in the multivariate regression model, with the exception of the following (based on consultation with sponsor team) for hospital-acquired: culture site and sepsis or severe sepsis (23% of study population had severe sepsis). For community-acquired, the following were removed from the model: length of hospital stay prior to index date, antibiotic exposure prior to current admission, alcohol use, and respiratory disease.

Prior antibiotic use was run as both a continuous and categorical/indicator variable, and the decision was made to use continuous variable. Age, sex, and race were removed from the final model. The following comorbidities were excluded in the analyses: connective tissue disease-rheumatic disease, peptic ulcer disease, moderate or severe liver disease, metastatic carcinoma, AIDS/HIV. Because the database only included month and year of admission, we assumed that all other (ie, non–infection-related) admissions that occurred during the month of the “qualifying” admission occurred prior to that admission. Thus, for example, a patient with an admission for myocardial infarction (MI) and another admission during which a cUTI was identified would be assumed to have developed the cUTI subsequent to discharge for the MI.

Because “dialysis” and “other renal failure” are almost 100% correlated (among 8696 patients who had chronic renal failure in the model training dataset, 99.2% [8626] had dialysis) and in order to have mutually excluded patients, only the dialysis variable was included in the final model; even “other renal failure” is also independently associated with CRE.

Highly correlated variables were evaluated on a case-by-case basis to determine whether they should be combined or excluded. Local CRE prevalence rate at the hospital level was also entered as an important predictor in the multivariate logistic regression model. This makes a strong assumption that CRE prevalence is constant over time. It also incorporates future data into the prediction: the probability of CRE for a patient in 2011 is based on future prevalence of CRE in 2014.

Summary statistics for prevalence were calculated, including mean, median, and range. Depending on distribution, prevalence was dichotomized at the median prevalence for all hospitals for all years. The CRE prevalence rate was dichotomized at 2% based on the overall distribution of the study sample.

The LASSO technique was used to select a set of risk factors that provide the best predictive accuracy for Gram-negative resistance and Gram-negative specific resistant pathogens (CRE, 3GC-R, and MDRP). The LASSO technique minimized some coefficients and set the coefficients of the least significant variables to 0 to reduce the variance and improve the model accuracy and interpretation. The logit function based on the coefficients of the set of final predictors in the LASSO model was used to estimate the probability of having or developing a Gram-negative resistant infection and separate models for the probability of having CRE, 3GC-R, and MDRP (4 separate models total).

The study sample was randomly split into the training set (70% of the study sample) and the test set (30% of the study sample). The training set was used to construct the LASSO logistic regression model. Cross-validation based on area under the curve (AUC) of receiver operating characteristic (ROC) curves was used to determine the best LASSO parameters and the final model. To accomplish cross-validation, the training set was partitioned into five subsets, one of which was selected to be the “validation set.” The remaining four sets were used as data for the model. The procedure was repeated 5 times, allowing each of the subsets to be used as the “validation set” once only. The AUC from each of the subsets was generated and averaged to provide the best LASSO parameter. The test set was used to validate the LASSO logistic regression model.

Model performance was evaluated by the measure of model lift and area under the ROC curve, (ie, correct prediction of target event among top 10% scored subjects and lift of top 10% scored subjects). Model accuracy was evaluated by true negative (specificity) and true positive (sensitivity) in the training and test sets. The glmmLasso package in R (<https://www.r-project.org/>) was used to perform the LASSO logistic regression. All calculations were performed using R.

### Sensitivity analyses

Approximately 21% of CRE patients and 8% of non-CRE patients had “transfer” as their admission code, but the reason for their transfer was unknown. It is possible that patients were transferred because they were considered high-risk for CRE. To evaluate whether transfer status had a confounding effect, the analysis was run in each subgroup of transfers and nontransfers. This sensitivity analysis was conducted for CRE, 3GC-R, and MDRP.

Supplementary Table S1. Baseline Event Count

| **Event Type** | **Total Patients Infected, %** | **Study Population (N=124,068)** | | | |
| --- | --- | --- | --- | --- | --- |
|  |  | **Training Dataset, n** | | **Test Dataset, n** | |
|  |  | **Infected** | **Not Infected** | **Infected** | **Not Infected** |
| **CRE** | 1.90 | 1323 | 67,991 | 553 | 28,950 |
| **3GC-R** | 12.09 | 8917 | 64,185 | 3696 | 27, 531 |
| **MDRP** | 3.91 | 2792 | 69,566 | 1244 | 29,654 |

3GC-R=third-generation cephalosporin-resistant Enterobacteriaceae; CRE=carbapenem-resistant Enterobacteriaceae; MDRP=multidrug-resistant *Pseudomonas aeruginosa*.

Supplementary Table S2. Predictors and Coefficients Included in Multivariate Regression Models

|  |  |  | | Coefficients* | |  | |
| --- | --- | --- | --- | --- | --- | --- | --- |
|  |  | CRE | | 3GC-R | | MDRP | |
| Predictor | Level^†^ | Hospital | Community | Hospital | Community | Hospital | Community |
| Intercept |  | –8.5735 | –7.4076 | –6.4249 | –4.6391 | –7.0473 | –6.3075 |
| cUTI | Yes | 0 | 0.6840 | 0 | 0.6123 | 0 | 0.7947 |
| cIAI | Yes | 0.7649 | 0 | 0.9268 | 0 | 0 | 0 |
| HAP/VAP | Yes | 1.0772 | 0.6953 | 0.6963 | 0 | 1.4314 | 2.2047 |
| BSI | Yes | 0.641 | 0.5943 | 0.2686 | 0.3800 | 0 | 0 |
| Prior admission in last 6 months | Yes | 0.2376 | 0.4355 | 0.3609 | 0.4761 | 0.3730 | 0.6599 |
| Prior infection in last 3 months | Yes | 0 | 0.7162 | 0 | 0.6649 | 0.7585 | 1.1228 |
| Prior # of antibiotics in current admission | 2–3 | 2.1028 | 0 | 2.0336 | 0 | 2.3256 | 0 |
| Prior # of antibiotics in current admission | 4+ | 2.6498 | 0 | 2.4129 | 0 | 3.8098 | 0 |
| Hospital unit | ICU | 0.4208 | 0 | 0.5417 | 0 | 0.1664 | 0 |
| Admitting source | Transfer | 0.2947 | 0.565 | 0.4231 | 0.3547 | 0.4971 | 0.5751 |
| Age category, y | 26–35 | 0 | 0 | 0.6593 | 0.4388 | 0 | 0 |
|  | 36–45 | 0 | 0 | 0.8667 | 0.7091 | 0 | 0 |
|  | 46–55 | 0 | 0 | 0.9369 | 0.8444 | 0 | 0 |
|  | 56–65 | 0 | 0 | 1.0076 | 0.9489 | 0 | 0 |
|  | 65+ | 0 | 0 | 0.8162 | 0.8724 | 0 | 0 |
| Hospital prevalence ≥ median | Yes | 1.9383 | 2.1848 | 0.8418 | 0.8325 | 1.0086 | 0.812 |
| Dialysis | Yes | 0.4475 | 0.2667 | 0.376 | 0.2262 | 0.1749 | 0 |
| Diabetes with complications | Yes | 0.6334 | 0 | 0.3423 | 0.1702 | 0 | 0 |
| Diabetes without complications | Yes | 0 | 0.4121 | 0 | 0.1848 | 0 | 0.216 |
| Peripheral vascular disease | Yes | 0 | 0 | 0.1735 | 0 | 0 | 0 |
| Paraplegia and hemiplegia | Yes | 0 | 0.6683 | 0 | 0.5699 | 0.7326 | 1.2115 |
| Chronic pulmonary disease | Yes | 0 | 0 | 0 | 0 | 0.2109 | 0.1418 |
| Myocardial infarction | Yes | 0.3233 | 0 | 0 | 0 | –0.3159 | –0.3169 |
| Cancer | Yes | 0 | 0 | 0.2295 | 0 | 0 | –0.3705 |
| Cerebrovascular disease | Yes | 0.3579 | 0 | 0.2748 | 0 | 0 | 0 |
| Congestive heart failure | Yes | 0 | 0 | 0.1846 | 0 | 0 | 0 |
| Mild liver disease | Yes | 0.3362 | 0 | 0.235 | 0 | 0 | 0 |

3GC-R=third-generation cephalosporin-resistant Enterobacteriaceae; BSI=bloodstream infection; cUTI=complicated urinary tract infection; cIAI=complicated intra-abdominal infection; CRE=carbapenem-resistant Enterobacteriaceae; HAP/VAP=hospital-acquired/ventilator-associated pneumonia; MDRP=multidrug-resistant *Pseudomonas aeruginosa*.

*A value of 0 for a predictor indicates that the predictor was not included in that model based on the results of the multivariate analysis.

^†^For yes/no predictors, a value of “no” is taken as the reference value and confers no additional risk. For prior number of antibiotics, 0–1 antibiotic is the reference level, and for age, the 18- to 25-year group is the reference.

**Supplemental Table S3. TRIPOD Checklist Prediction Model Development**

| **Section/Topic** | **Item** | **Checklist Item** | **Page** |
| --- | --- | --- | --- |
| **Title and abstract** | | | |
| Title | 1 | Identify the study as developing and/or validating a multivariable prediction model, the target population, and the outcome to be predicted. | 1 |
| Abstract | 2 | Provide a summary of objectives, study design, setting, participants, sample size, predictors, outcome, statistical analysis, results, and conclusions. | 2–3 |
| **Introduction** | | | |
| Background and objectives | 3a | Explain the medical context (including whether diagnostic or prognostic) and rationale for developing or validating the multivariable prediction model, including references to existing models. | 4–5 |
|  | 3b | Specify the objectives, including whether the study describes the development or validation of the model or both. | 5–6 |
| **Methods** | | | |
| Source of data | 4a | Describe the study design or source of data (eg, randomized trial, cohort, registry data), separately for the development and validation datasets, if applicable. | 5–6 |
|  | 4b | Specify the key study dates, including start of accrual, end of accrual, and, if applicable, end of follow-up. | 6 |
| Participants | 5a | Specify key elements of the study setting (eg, primary care, secondary care, general population), including number and location of centers. | 5–6 |
|  | 5b | Describe eligibility criteria for participants. | 6–7 |
|  | 5c | Give details of treatments received, if relevant. | 7 |
| Outcome | 6a | Clearly define the outcome that is predicted by the prediction model, including how and when assessed. | 10 |
|  | 6b | Report any actions to blind assessment of the outcome to be predicted. | NA |
| Predictors | 7a | Clearly define all predictors used in developing or validating the multivariable prediction model, including how and when they were measured. | 7–8, Suppl. Materials |
|  | 7b | Report any actions to blind assessment of predictors for the outcome and other predictors. | NA |
| Sample size | 8 | Explain how the study size was arrived at. | 5, 9 |
| Missing data | 9 | Describe how missing data were handled (eg, complete-case analysis, single imputation, multiple imputation) with details of any imputation method. | Suppl. Materials |
| Statistical analysis methods | 10a | Describe how predictors were handled in the analyses. | 9–10 |
|  | 10b | Specify type of model, all model-building procedures (including any predictor selection), and method for internal validation. | 9–10 |
|  | 10d | Specify all measures used to assess model performance and, if relevant, to compare multiple models. | 9–10 |
| Risk groups | 11 | Provide details on how risk groups were created, if done. | Suppl. Materials |
| **Results** | | | |
| Participants | 13a | Describe the flow of participants through the study, including the number of participants with and without the outcome and, if applicable, a summary of the follow-up time. A diagram may be helpful. | 10–11 |
|  | 13b | Describe the characteristics of the participants (basic demographics, clinical features, available predictors), including the number of participants with missing data for predictors and outcome. | 10–11 |
| Model development | 14a | Specify the number of participants and outcome events in each analysis. | 10–11, Suppl. Table S1 |
|  | 14b | If done, report the unadjusted association between each candidate predictor and outcome. | Suppl. Materials |
| Model specification | 15a | Present the full prediction model to allow predictions for individuals (ie, all regression coefficients, and model intercept or baseline survival at a given time point). | Suppl. Table S2 |
|  | 15b | Explain how to the use the prediction model. | 13, Suppl. File 2, Fig 1, |
| Model performance | 16 | Report performance measures (with CIs) for the prediction model. | Table 2 |
| **Discussion** | | | |
| Limitations | 18 | Discuss any limitations of the study (eg, nonrepresentative sample, few events per predictor, missing data). | 17–18 |
| Interpretation | 19b | Give an overall interpretation of the results, considering objectives, limitations, and results from similar studies, and other relevant evidence. | 13–18 |
| Implications | 20 | Discuss the potential clinical use of the model and implications for future research. | 13–14, 18–19 |
| **Other information** | | | |
| Supplementary information | 21 | Provide information about the availability of supplementary resources, such as study protocol, Web calculator, and datasets. | 21, Suppl file 1 and 2 |
| Funding | 22 | Give the source of funding and the role of the funders for the present study. | 21 |
